# Models of knot and stem development in black spruce trees indicate a shift in allocation priority to branches when growth is limited

**DOI:** 10.7717/peerj.873

**Published:** 2015-04-09

**Authors:** Emmanuel Duchateau, David Auty, Frédéric Mothe, Fleur Longuetaud, Chhun Huor Ung, Alexis Achim

**Affiliations:** 1 Renewable Materials Research Centre, Université Laval, Québec, QC, Canada; 2 AgroParisTech, UMR1092 LERFoB, Nancy, France; 3 Natural Resources Canada, Canadian Wood Fibre Centre, Laurentian Forestry Centre, Sainte-Foy, Québec, Canada

**Keywords:** Milton’s law, Knot morphology models, Resource partitioning, X-ray computed tomography, *Picea mariana*, Branch autonomy principle

## Abstract

The branch autonomy principle, which states that the growth of individual branches can be predicted from their morphology and position in the forest canopy irrespective of the characteristics of the tree, has been used to simplify models of branch growth in trees. However, observed changes in allocation priority within trees towards branches growing in light-favoured conditions, referred to as ‘Milton’s Law of resource availability and allocation,’ have raised questions about the applicability of the branch autonomy principle. We present models linking knot ontogeny to the secondary growth of the main stem in black spruce (*Picea mariana* (Mill.) B.S.P.), which were used to assess the patterns of assimilate allocation over time, both within and between trees. Data describing the annual radial growth of 445 stem rings and the three-dimensional shape of 5,377 knots were extracted from optical scans and X-ray computed tomography images taken along the stems of 10 trees. Total knot to stem area increment ratios (KSR) were calculated for each year of growth, and statistical models were developed to describe the annual development of knot diameter and curvature as a function of stem radial increment, total tree height, stem diameter, and the position of knots along an annual growth unit. KSR varied as a function of tree age and of the height to diameter ratio of the stem, a variable indicative of the competitive status of the tree. Simulations of the development of an individual knot showed that an increase in the stem radial growth rate was associated with an increase in the initial growth of the knot, but also with a shorter lifespan. Our results provide support for ‘Milton’s Law,’ since they indicate that allocation priority is given to locations where the potential return is the highest. The developed models provided realistic simulations of knot morphology within trees, which could be integrated into a functional-structural model of tree growth and above-ground resource partitioning.

## Introduction

Models of carbon assimilate allocation in trees generally consider branches to be part of either the woody shoot or the crown (Landsberg & Waring, 1997; Mathieu et al., 2009). However, considering branch xylem as a separate sink can extend the practical applicability of functional-structural tree models (FSTMs; Sievänen et al., 2000) to include wood properties considerations. Knots are formed when branches are occluded by growing tree stems, and exert a strong influence on the end-use characteristics of wood products (Buksnowitz et al., 2010).

Knot formation is driven by complex spatiotemporal interactions between a tree and its environment. Thus, knowledge of the biological processes that regulate assimilate partitioning in trees could improve models of branch growth. The branch autonomy principle (Van der Wal, 1985; Sprugel & Hinckley, 1988) has been used in some FSTMs to simplify the modelling process (Bosc, 2000; Kull & Tulva, 2000). The branch autonomy principle states that the growth of individual branches can be predicted from their morphology and position in the forest canopy, irrespective of tree characteristics. Models that incorporate this principle can also predict mortality based on the growing space (Mitchell, 1975) or the amount of light (Nikinmaa & Hari, 1990) available to individual branches. However, there is an important limitation to this principle. By comparing the height of the lower limit of the living crown in trees of different sizes, Sprugel (2002) showed that branches on supressed trees were more likely to survive and grow than the equivalent branches on dominant trees. This implied shift in allocation priority within trees towards branches in light-favoured positions, referred to as ‘Milton’s Law of resource availability and allocation’ (Sprugel, 2002), suggests that assimilates are invested where the potential return is highest. This is consistent with the results of Nikinmaa et al. (2003), who obtained improved predictions of crown development when considering both the position and the light environment of branches. However, experimental confirmation of Milton’s Law is generally restricted to static assessments of the location of the crown base in even-aged forest stands (Valentine et al., 2013).

Branch ontogeny can be studied in long-term experiments (Pretzsch, 2005), but repeated measurements on the same trees are time-consuming and costly. One solution to this problem is to use empirical branch distribution models to simulate the temporal development of tree and branch growth using cross-sectional data i.e., observations of the number, location and size of branches made on trees of different ages (Colin & Houllier, 1991; Mäkinen & Mäkelä, 2003; Achim et al., 2006; Weiskittel, Maguire & Monserud, 2007). However, the simplicity of the approach comes at the expense of reduced accuracy for some branch measurements (Duchateau et al., 2013a). More recently, non-destructive techniques for rapidly generating high-resolution data have been developed, such as infrared imaging, optical scanning, magnetic resonance imaging (MRI), and computed tomography (CT) using X-rays or gamma rays (Moberg, 2001; Longuetaud et al., 2012; Dutilleul, Han & Beaulieu, 2014). These innovations allow the use of internal data to simultaneously reconstruct stem and knot growth over time.

In this study we present models linking knot ontogeny to the secondary growth of the main stem in black spruce (*Picea mariana* (Mill.) B.S.P.), a dominant species in the North American boreal forest. We used data from high-resolution CT scans of tree stems to reconstruct the history of both stem and knot development, with the aim of developing models that would apply in an FSTM framework. First, we tested the hypothesis that the ratio of branch to stem growth was dependent on stem characteristics indicative of the competitive status of the tree. We then developed statistical models for predicting the evolution of individual knot diameter and trajectory using a series of predictors related to the position in the tree, stem radial growth, and other general stem characteristics. This allowed us to test ‘Milton’s Law’ using longitudinal data i.e., repeated measurements of branch and stem growth over time. This approach allowed us to make detailed simulations of knot development while considering the variation in assimilate partitioning between trees.

## Materials and Methods

### Tree sampling

Sample trees were collected from seven naturally-regenerated, unmanaged forest stands in the North-Shore region of Quebec, Canada. All sampling locations were part of a network of sites established to study the growth of spruce-moss forests after fire (Barrette, Pothier & Ward, 2013; Torquato et al., 2014; Ward, Pothier & Paré, 2014). At the time these plots were established, efforts were made to maintain site characteristics (i.e., surface deposit, topographic position, exposure and soil drainage) as constant as possible and representative of mesic conditions (Ward, Pothier & Paré, 2014).

Because CT scanning is costly and the associated data processing time-consuming, we worked with a limited number of sample trees. In each of the seven stands, two trees were randomly selected for destructive sampling. However, four trees were omitted from the analysis due to missing discs and the presence of wood decay. Of the ten trees in our final sample, eight came from even-aged plots that had regenerated after fires dating back to between 66 and 152 years (Bouchard, Pothier & Gauthier, 2008). Two more trees (T09 and T10) were selected from one uneven-aged plot where the time since the last stand-replacing fire exceeded 200 years. The sample trees had a wide range of ages, crown size and stem dimensions (Table 1).

**Table 1:** Characteristics of the 10 sample trees in the dataset.

	Age	Number of complete rings used on the analysis	Total height (m)	Diameter at breast height (cm)	Length of the crown^a^ (m)	Number of measured knots
**T01**	82	14	14.02	15.4	5.04	726
**T02**	85	19	14.15	14.1	4.3	620
**T03**	86	27	15.27	15.6	4.8	819
**T04**	93	32	11.81	14.3	2.09	568
**T05**	104	45	14.22	16.3	5.32	1,066
**T06**	106	47	20.52	22.2	8.77	1,198
**T07**	113	48	18.2	21.4	5.82	514
**T08**	118	51	16.92	21.8	8.32	1,121
**T09**	139	78	16.28	17.8	5.42	993
**T10**	152	84	20.8	22.4	5.25	1,518
**Mean**	107.8	68.5	16.219	18.13	5.513	914.3
**Sd**	23.47	24.36	2.93	3.45	1.90	321.14

**Notes.**

a The base of the crown was defined as the location of the lowest pseudo-whorl containing at least one live branch, above which all pseudo-whorls contained at least one live branch.

### Annual knot data

After felling, each tree was cut into 2.5-m logs, giving a total of 41 logs that were then transported to the Institut National de la Recherche Scientifique in Quebec City and scanned using a Somatom Sensation 64 CT scanner (Siemens Medical Solutions USA, Inc., Malvern, Pennsylvania, USA). Each log was scanned at 2-mm intervals along its longitudinal axis with a 2-mm-wide X-ray beam (120 kV–50 mA), so that the scanned segments were contiguous. The pixel size was 0.35 mm × 0.35 mm in the transverse direction.

**Figure 1:**
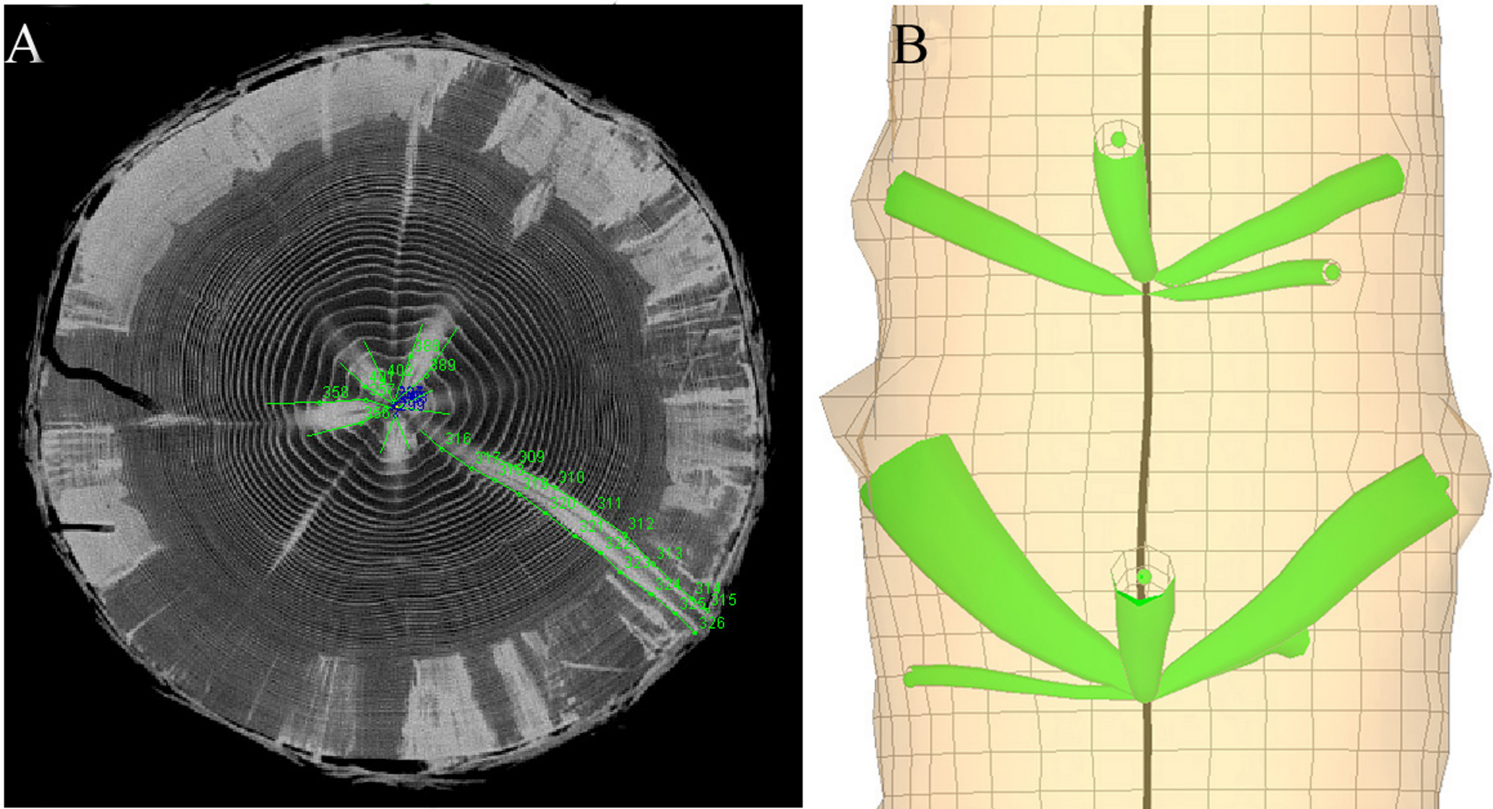
The knot extraction process. (A) Extraction of the position and diameter of each knot profile on CT scanning images using the ImageJ Java plug-in ‘Gourmand’ and (B) reconstruction of the 3D geometry of each knot using the software “Bil3D.”

Knot geometry was extracted using the ImageJ 1.44 free software (Abramoff, Magalhaes & Ram, 2004), with a Java plug-in (‘Gourmand,’ version 1.01) developed at INRA, Nancy, France (Longuetaud et al., 2012). On successive images, the tangential limits of each knot were manually delineated with a series of points (Fig. 1A). A second purpose-built software named ‘BIL3D’ (Colin et al., 2010) was developed to visualise the position and 3D geometry of each knot using the Cartesian coordinates of each point (Fig. 1B). The series of points representing the tangential limits of the knot were interpolated using spline curves. This allowed us to position the central axis (as the middle of both curves) and diameter (as the distance between each curve, assuming a circular cross section) of each knot from its point of origin to the bark. In a database, the diameter (*D*) of the knot was recorded at an interval of 1 cm from the stem’s pith in the radial direction. Similarly, the position of the central axis of the knot along the longitudinal stem axis (*Z*, referred to as the ‘trajectory’) was recorded at an interval of 1 cm from the stem’s pith. This way, we obtained a representation of the geometric profiles of 5,377 knots. A more detailed description of the knot reconstruction method was presented by Duchateau et al. (2013a).

The demarcation between stem and knot xylem cannot be considered as perfectly discrete. Knot profiles were therefore extracted from the CT images by manually delineating high density wood corresponding to a knot and the surrounding lower density stem wood. Although the transition was generally clear enough to ensure accuracy (Fig. 1), the knot reconstruction process produced some localized irregularities that did not reflect the true shape of the knots. For this reason, we chose to smooth the radial profiles of each knot using a combination of two Weibull equations, which can reproduce a wide variety of knot profiles (Duchateau et al., 2013a). This also had for advantage to provide a parametric description of each knot that was dependent on the radial position within the stem. It is possible, however, that abrupt variations in knot shape were missed due to the smoothing process.

Knot development at a given radial position (*l*) was reconstructed using the diameter (*D_l_*) and trajectory data (*Z_l_*). The same Weibull equation with an additional linear term was used to model both series of *D_l_* and *Z_l_* measurements:

(1) \begin{eqnarray*} {y}_{l}=\alpha \left(1-{e}^{\left(-\beta \left(\frac{1}{{R}_{\max }-l}\right)\right)}\right)+\mu \cdot l\hspace{1em}\hspace{1em}(0\leq l\lt {R}_{\max }) \end{eqnarray*}

where *y_l_* represents either the *D_l_* or *Z_l_* values (mm), *l* is the distance from the stem’s pith in the radial direction (mm), *R*_max_ is the total length (mm) of the knot along the stem’s radial direction and *α*, *β* and *μ* are parameters to be estimated empirically.

The functions were fitted to each knot independently using the *nls* function of the *nlme* library in the R statistical programming environment (R Core Team, 2014). The models for both *D_l_* and *Z_l_* converged for 95% of the knots in the database. Visual examination revealed that non-convergent knots were generally small and sinuous. Indeed, convergent knots represented 98% of the total volume of knots in the entire dataset, which we considered representative of the full history of knot growth in our sample trees.

### Annual ring data from the main stem

The model presented by Duchateau et al. (2013a) only made static predictions of knot shape based on external branch characteristics. To meet the objective of this study to link knot ontogeny to the secondary growth of the main stem, it was necessary to reconstruct the yearly growth of the stem at its interface with each knot. Annual ring data from the main stem were difficult to obtain from the CT images due to factors such as narrow rings and the higher moisture content of the sapwood. One-cm-thick discs were hence cut from the ends of each log to reconstruct the growth history of the stems. Discs were optically scanned and annual ring boundaries were delineated in the four cardinal radial directions using image analysis software (WinDENDRO™; Régent Instruments, Quebec City, Quebec, Canada, 2005; Guay, Gagnon & Morin, 1992).

**Figure 2:**
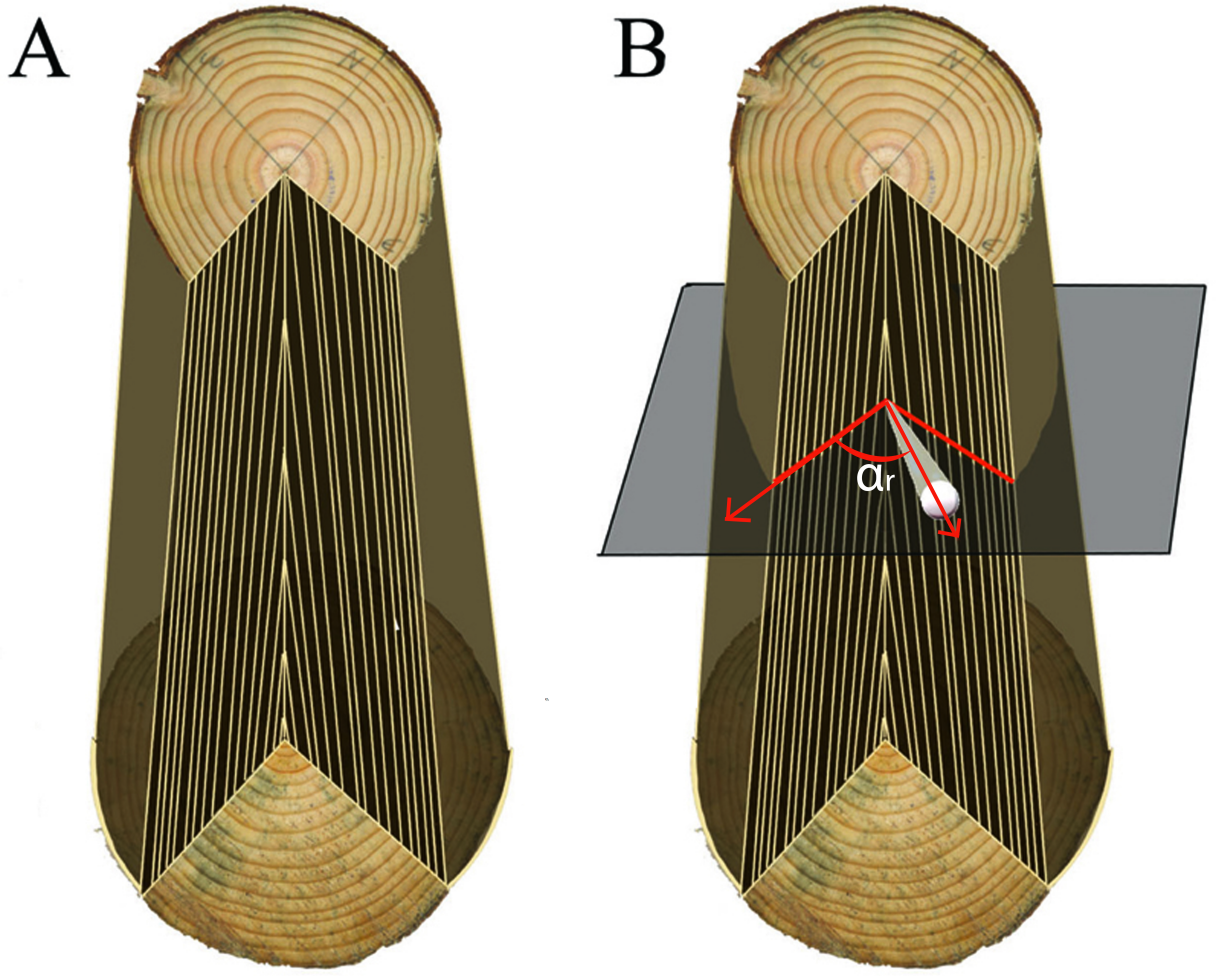
Inferring ring width at the location of a knot. (A) Interpolation of the rings between the two discs to reconstruct the log and (B) selection of the two cardinal directions bordering the knot to reconstruct the ring widths along the knot profile.

To link annual changes in knot geometry with stem radial increments, a first linear interpolation was made, in each cardinal direction, between the widths of each matching ring from both ends of each log (Fig. 2A). For rings present near the pith of the lower disc but absent from the upper disc, we used the mean slope and intercept of linear interpolations derived for the first five complete rings. This way, we obtained estimates of annual ring widths at any height along the stem in the four main cardinal directions.

To obtain estimates of stem growth in the azimuthal direction of a knot (Fig. 2B), a second interpolation was made from the two surrounding cardinal directions for which we had annual ring width measurements. In this case we used a weighted average of the two known ring width series located on each side of the knot. We defined *α_r_* as the azimuth angle between a knot and one of the two cardinal directions on each side. The weighting factor was calculated as (90-*α_r_*)/90, which approached a value of 1 if the knot orientation was close to one of the two cardinal directions. Due to irregularities in stem shape, the resulting series of stem rings associated with a given knot did not end in the same exact location as the knot-stem interface, which was located on the CT images. Therefore, a small correction constant was added (or subtracted) to each ring in the series to ensure that both matched exactly. These linear interpolations of annual ring width variation between two sample discs were a simplification, since in reality growth rings deviate around knots (Pellicane & Franco, 1994). However, given the imposibility to extract the position of growth rings along each knot directly from CT images, this was considered as a good approximation.

In a final step in the knot and stem growth reconstruction process, we traced back the annual limits of primary growth. Each annual elongation of the shoot was defined as a growth unit (GU). Like other conifers, black spruce produces several nodal and internodal branches within a growth unit. Nodal branches are those forming a whorl at the top of a GU (Achim et al., 2006; Auty et al., 2012). Botanically, the branches of conifers do not technically originate from the same vertical position, these are referred to as ‘pseudo-whorls’ (Fisher & Honda, 1979). However, this distinction was not apparent at the resolution of our CT-scanning measurements. Therefore, we summed the basal areas of all branches that originated from the same CT image, which facilitated the identification of pseudo-whorls of branches that were used as the limits of annual GUs. To avoid large errors, we ensured that the number of GUs matched the difference in the number of annual rings measured at both ends of each log. A more detailed description of the growth unit identification method is presented in Duchateau et al. (2013b).

**Figure 3:**
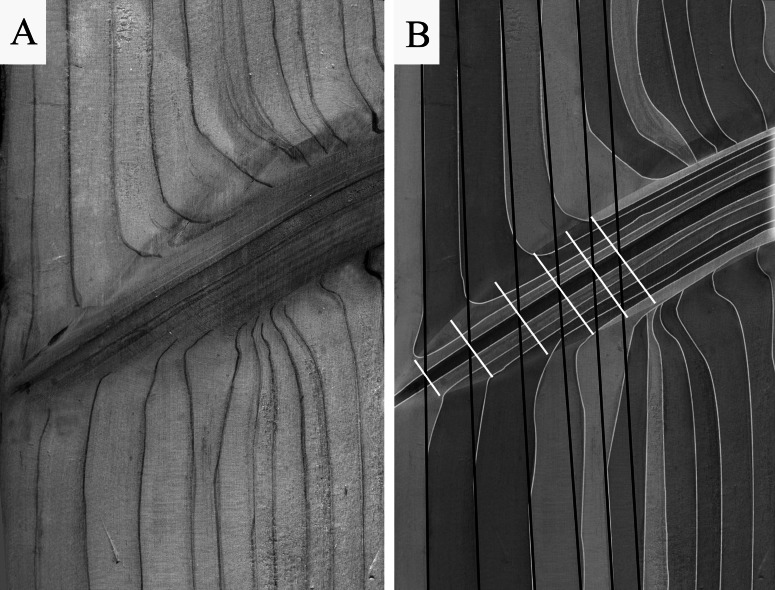
Inferring knot annual increments. (A) Example of ring width deformations around a knot; (B) extraction of the annual knot data.

Once we had obtained a full description of both the knots and stem shape, a final step was to obtain the annual increments in knot diameter (Δ*D_t_*) and trajectory (Δ*Z_t_*). These were computed using the intersection points between stem rings and knots, and by considering the diameter perpendicular to the central axis of the knot at each intersection point (Fig. 3).

### Model development

#### Tree-level models

To examine the variation in biomass allocation between the stem and branches over time, the ratio of knot to stem growth (KSR_*i*,*t*_, dimensionless) was calculated, for each year of growth (*t*) in a tree, as the sum of all knot area increments at the surface of the stem divided by the annual basal area increment of the stem at 1.3 m. Because the trees were not scanned all the way to the stem apex, the most recent annual growth rings were incomplete. These were therefore omitted from the analysis so that calculations were made only for years where complete growth data were available. When knots had reached a constant or decreasing diameter they were considered to be dead.

To assess the variation of KSR_*i*,*t*_ through the life of the tree, we developed a linear mixed-effects model (Pinheiro & Bates, 2009) describing its evolution as a function of tree height-diameter ratio and tree age. To assess the effect of within stand competition on KSR_*i*,*t*_, the ratio (HD_*i*,*t*_, m/cm) between tree height (H_*i*,*t*_) and its diameter at breast height (DBH_*i*,*t*_, measured at 1.3 m) was used as a surrogate for the competitive status of the subject trees at a given age. This ratio is useful because inter-tree spacing is known to strongly affect crown development and hence the radial growth of the stem, whereas it has much less effect on height growth (Weiskittel et al., 2011). Since values of KSR_*i*,*t*_ were continuous and non-negative, it was modelled as a gamma distribution with a log-link:

(2) \begin{eqnarray*} \ln (\mathrm{KSR})_{i,t}={a}_{1}+{a}_{2}\cdot {\mathrm{HD}}_{i,t}+{a}_{3}\cdot {\mathrm{Age}}_{i,t}+{\delta }_{i}+\varepsilon \end{eqnarray*}

where ln(KSR_*i*,*t*_) is the natural logarithm of the knot to stem ratio in a given year *t*, Age_*i*,*t*_ is the age of the tree (years), *a*_1_, *a*_2_, *a*_3_ are the model parameters, *δ_i_* is the random effect for each tree (*i*), and ε is the residual error of the model.

Next, we examined the effect of KSR_*i*,*t*_ on the number of new branches produced in a given year by fitting a Poisson regression model, with a log-link, describing the number of new branches per stem as a function of KSR_*i*,*t*_, tree age and their interaction:

(3) \begin{eqnarray*} \ln ({\mathrm{NBR}}_{i,t})={b}_{1}+{b}_{2}\cdot {\mathrm{KSR}}_{i,t}+{b}_{3}\cdot {\mathrm{Age}}_{i,t}+{b}_{4}\cdot {\mathrm{KSR}}_{i,t}\cdot {\mathrm{Age}}_{i,t}+{\delta }_{i}+\varepsilon \end{eqnarray*}

where ln(NBR_*i*,*t*_) is the natural logarithm of the number of new branches per stem in a given year, *b*_1_, *b*_2_, *b*_3_, *b*_4_ are the model parameters, and all other variables are as previously defined.

The models presented in Eqs. (2) and (3) were fitted using the *glmer* function in the *lme4* library (Bates et al., 2014) of the R statistical programming environment (R Core Team, 2014). In model fitting, we began by screening all potential tree-level explanatory variables and biologically plausible interaction terms. Variables were selected after calculating the variance inflation factors (VIF), to address any potential multicollinearity issues (O’brien, 2007). Variables that were highly correlated (VIF > 4) were excluded from the models. Variable selection for Eqs. (2) and (3) was the result of a backwards elimination process in which the selection was based on Akaike’s information criterion (AIC) (Akaike, 1974). Chi-squared-based likelihood ratio tests were used to evaluate the significance of terms that were successively dropped from the model. In the absence of a significant difference (*p* > 0.05), the simplest model was retained. Parameter estimates were obtained using the maximum likelihood method.

#### Individual knot models

Next, statistical models were developed to describe the temporal evolution of the morphology of individual knots using annual ring- and tree-level characteristics as independent variables. Initially, we attempted to fit a single model describing both trajectory (*Z*_*i*,*j*,*t*_) and knot diameter (*D*_*i*,*j*,*t*_) simultaneously, thereby reconstructing the entire knot in a single step. However, this led to an underestimation of knot diameter in the first years of growth that carried over for the entire knot profile. Therefore, separate models were developed for each separate component. Individual knot diameter and trajectory models were fitted to the data from a random selection of 75% of the total population of knots, while the remaining data were used for model evaluation.

##### Knot diameter model

**Figure 4:**
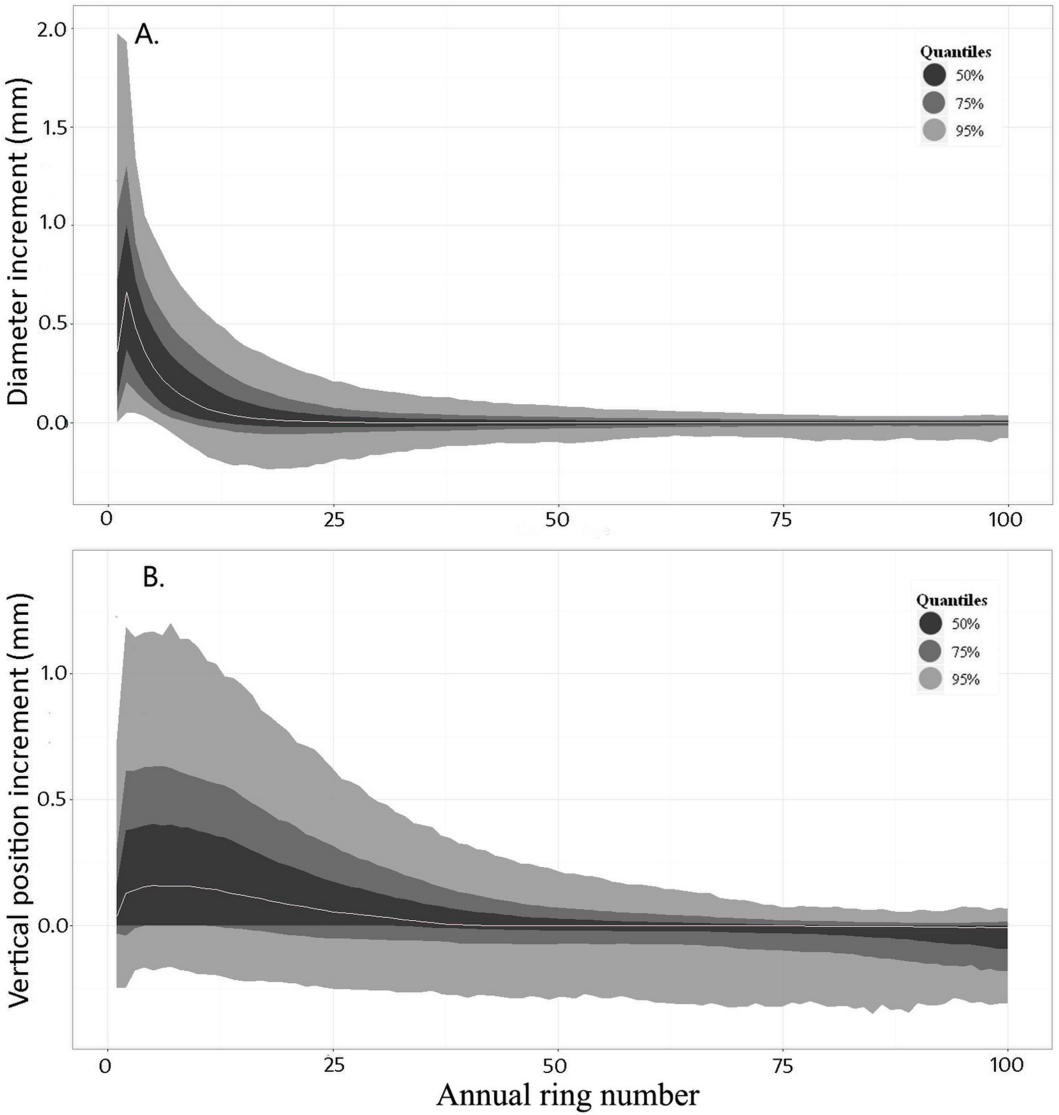
Distributions of annual increments in diameter (Δ*D_t_*) and trajectory (Δ*Z_t_*) of the knot against annual ring number from the stem’s pith. The grey line indicates the median of all observations for a given ring number. Contours provide the distribution quantiles around the median

We observed relatively consistent patterns in the diameter development of the knots. There was a rapid increase in diameter increment in the first three years of knot growth, followed by a gradual decline of growth until branch death (Fig. 4A). On average, branch increments reached zero at around year 25. We hence divided each diameter profile into three sections: (1) the initiation section (years 0–3), (2) the growth section (years 4–25) and (3) the stable or declining section (years > 25). In the initiation section, because Δ*D*_*i*,*j*,*t*_ values did not follow a Gaussian distribution, *D*_*i*,*j*,*t*_ was modelled directly. In the remaining two sections Δ*D*_*i*,*j*,*t*_ was used as the response variable.

Knot characteristics at time *t* − 1 were used to make predictions at time *t*. This ensured a smooth transition between the different sections of the model. After the variable selection process, the general form of the knot diameter model for each section was expressed as:

(4) \begin{eqnarray*} \Delta {D}_{i,j,t}~\text{or}~{D}_{i,j,t}={c}_{1}+{c}_{2}\cdot \Delta {D}_{i,j,(t-1)}+{c}_{3}\cdot {D}_{i,j,t-1}+{c}_{4}\cdot {\mathrm{GU}}_{\mathrm{pos}i,j}+{c}_{5}\cdot {l}_{i,j,t}+~{c}_{6}\cdot {\mathrm{RW}}_{i,j,t}+{c}_{7}\cdot {\mathrm{HD}}_{i,t}+{c}_{8}\cdot {\mathrm{Age}}_{i,t}+{c}_{9}\cdot {\mathrm{DBH}}_{i,t}+{c}_{10}\cdot {H}_{i,j}+{\delta }_{i}+{\delta }_{i,j}+\varepsilon \end{eqnarray*}

where GU_pos,*i*,*j*_ is the relative position of the knot initiation point along the GU (varies from 0 at the base to 1 at the stem apex, and is used to take the phenomenon of acrotony (Powell, 1995) into account), RW_*i*,*j*,*t*_ is the ring width of the stem at the location of the knot in year t, *δ_i_* and *δ*_*i*,*j*_ are the tree- and knot-level random effects and ε is the residual error. All other variables are as previously defined.

##### Knot trajectory model

The average annual variation of Δ*Z*_*i*,*j*,*t*_ was typically positive until approximately ring 40. After this point the trajectory stabilized, before decreasing after ring 60 (Fig. 4B). The knot trajectory profiles were therefore separated into two sections delineated at ring number 50. Characteristics of the knots in year *t* − 1 were also included in this model, thus ensuring a smooth transition between the sections. Various combinations of the explanatory variables were used in each section of the model. The general form of the knot trajectory model for each section was expressed as:

(5) \begin{eqnarray*} \Delta {Z}_{i,j,t}={d}_{1}+{d}_{2}\cdot {D}_{i,j,(t-1)}+{d}_{3}\cdot \Delta {Z}_{i,j,(t-1)}+{d}_{4}\cdot {l}_{i,j,t}+{d}_{5}\cdot {\mathrm{RW}}_{i,j,t}+{d}_{6}\cdot {\mathrm{GU}}_{\mathrm{pos}~i,j}+~{d}_{7}\cdot {\mathrm{HD}}_{i,t}+{d}_{8}\cdot {\mathrm{Age}}_{i,t}+{d}_{9}\cdot {\mathrm{DBH}}_{i,t}+{\delta }_{1}+{\delta }_{i,j}+\varepsilon \end{eqnarray*}

where all variables are as previously defined. See Table 2 or a full description of all variable names used in the models.

**Table 2:** Definitions and abbreviations of the variables used in this paper.

	Description
**Tree-level variables**	
DBH_*t*_	Diameter of the tree at 1.3 m at time *t* (mm)
Age_*t*_	Age of the tree at time *t*
HD_*t*_	Ratio of total tree height to DBH calculated for each year of growth at time *t*
KSR_*t*_	Ratio of total knot area increment to the stem basal area increment at time *t*
**Ring-level variables**	
RN	Annual ring number from the pith of the main stem at the level of each knot
RW_*t*_	Annual ring width at time *t* (mm)
*l_t_*	Distance from the pith of the stem at time *t* (mm)
GU_pos_	Relative position of the knot initiation point along the annual growth unit (varies from 0 to 1)
H_*k*_	Position of the initiation point of the knot along the stem (ground level = 0) (m)
**Knot-level variables**	
Δ*D_t_*	Annual increment of the knot diameter from time *t* − 1 to *t* (mm)
*D_t_*	Predicted knot diameter at time *t* (mm)
Δ*Z_t_*	Annual increment of the trajectory of the knot from time *t* − 1 to *t* (mm)

These models were fitted using functions contained in the *nlme* library of the R statistical programming environment (R Core Team, 2014). A power variance function of annual ring number from the pith at the level of each knot (RN) was included to account for heteroscedasticity in the model residuals. In addition, a continuous first-order auto-regressive term (AR1) was added to account for autocorrelation between successive measurements. The model fitting process started by including a full set of potential ring-, knot- or tree-level explanatory variables and model selection was performed using the same backwards elimination procedure as described in the section on tree-level models.

#### Simulations

To analyse the influence of tree growth and competitive status on knot development, we reconstructed a single knot at 6.1 m using the predictions from Eqs. (4) and (5) and the stem and growth characteristics of tree T10. Then, while keeping tree height constant, we increased the annual ring increments by 50%. The diameter and trajectory profiles of the original knot were then recalculated. The process was repeated by decreasing the annual stem increments of the same tree by 50% of their actual values and again predicting knot morphology.

In a second simulation, all knots from a 1.5-m section starting at a height of 2.5 m in tree T4 were simulated using Eqs. (4) and (5) and compared to the real knots, as extracted from the CT images. For this simulation we used the known insertion points along the stem and azimuthal orientation of each knot. Where appropriate, the year at which a knot was observed to be completely occluded by the growing stem was used as the end-point of the simulation.

## Results

### Tree-level models

**Figure 5:**
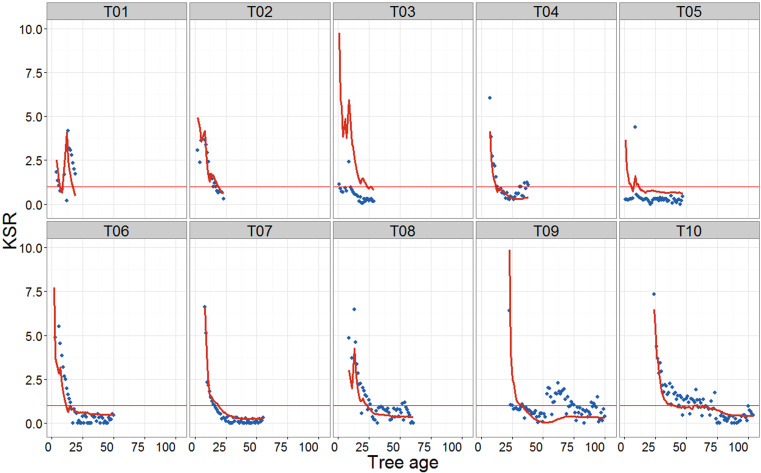
Scatterplots showing the evolution of KSR (total annual knot area increment/stem increment at 1.3 m) with tree age. Time series do not start at age 0 because HD_*t*_ assessments start when the stem has reached a height of 1.3 m. Points, observed values; red lines, model predictions (Eq. (2) and Table 3). Horizontal red line shows an equality between the total annual knot increment and the stem increment at 1.3 m (KSR = 1).

**Figure 6:**
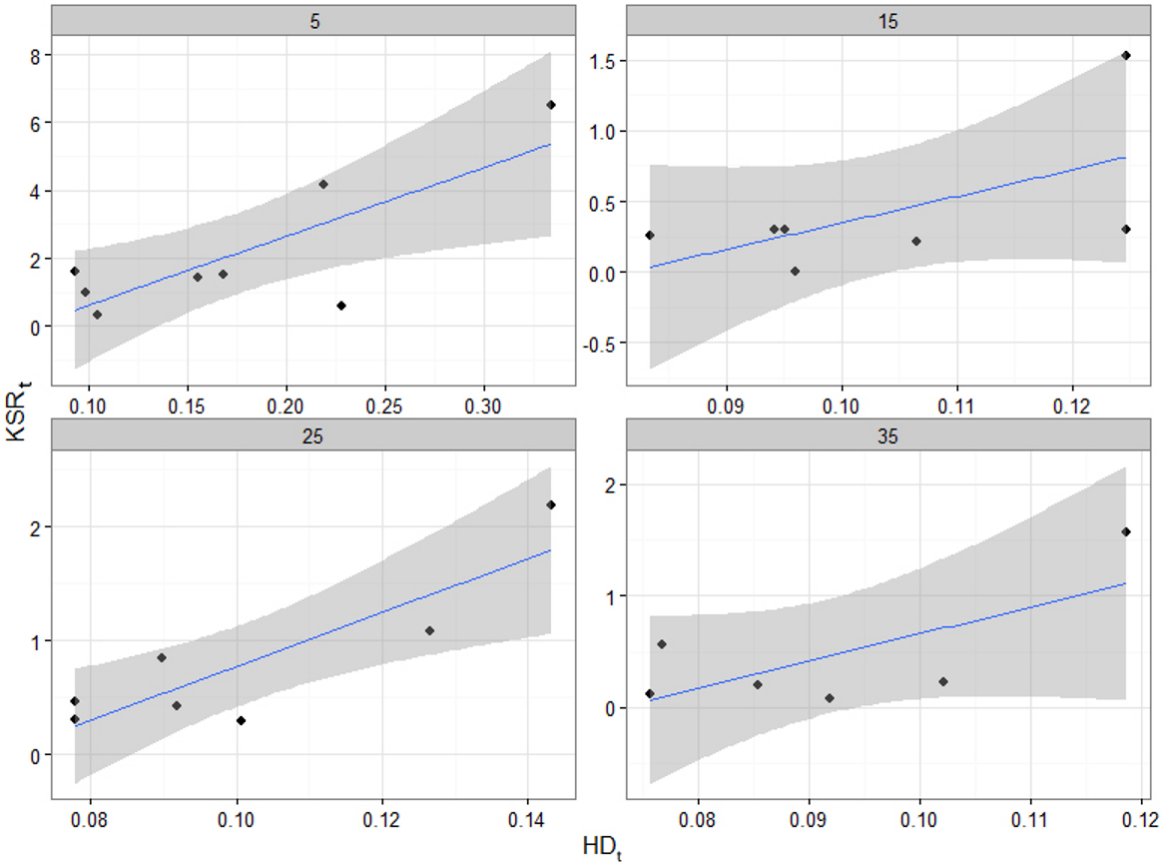
Scatterplots of observed KSR_*t*_ vs. HD_*t*_ in each sample tree for cambial ages 5, 15, 25 and 35 at breast height. The linear regressions fitted though the points show a positive correlation between the two variables for all ages. The shaded areas represent the standard errors.

The knot to stem increment ratio (KSR_*t*_) varied considerably with tree age. On average, KSR_*t*_ was higher when trees were young and decreased rapidly in the first few years, before stabilizing (Fig. 5). The rate of the initial decrease varied among trees. Values of KSR_*t*_ greater than 1 indicated that, in a given year, the total knot basal area increment exceeded that of the stem. In addition to the negative relationship with tree age, KSR_*t*_ ratio was positively related to HD_*t*_, such that more slender trees allocated relatively more biomass to their branches than to the main stem (Fig. 6). Furthermore, in a given year, the predicted number of new branches produced was greater in trees with higher KSR_*t*_ values, but the effect of KSR_*t*_ decreased with increasing tree age (Eq. (3) and Table 3).

**Table 3:** Fixed effects parameter estimates and standard errors of the KSR model given by Eq. (2) and the model for the number of new branches given by Eq. (3).

Model	Parameter	Estimate	S.E.	*P*-value
Equation (2)	*a*_1_	−0.3956	0.11947	<0.0001
	*a*_2_	4.1717	0.23896	<0.0001
	*a*_3_	−0.0114	0.00169	<0.0001
Equation (3)	*b*_1_	1.7864	0.15040	<0.0001
	*b*_2_	0.0354	0.00934	<0.0001
	*b*_3_	0.0153	0.00105	<0.0001
	*b*_4_	−0.0006	0.00024	<0.0001

**Figure 7:**
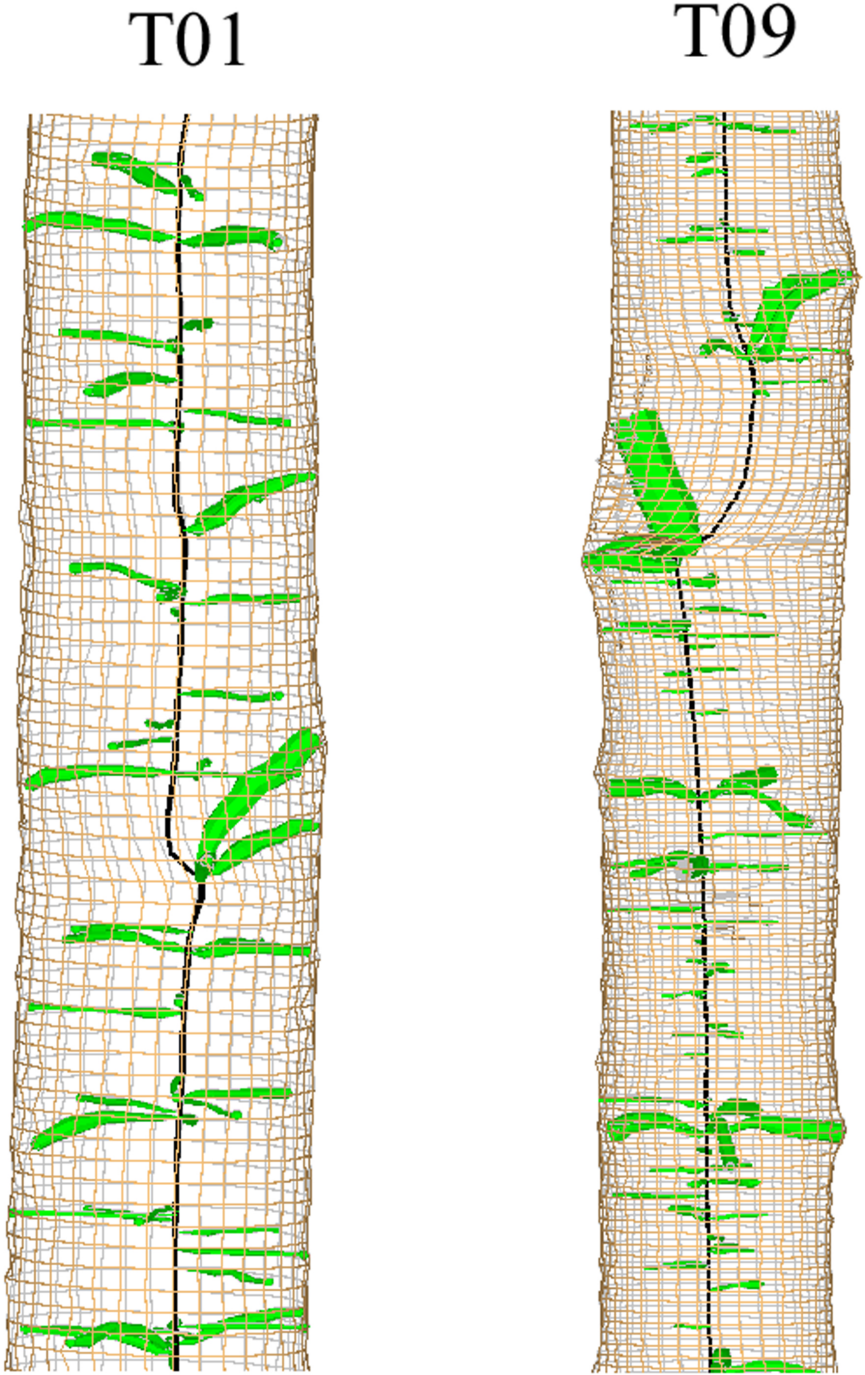
3D reconstruction of sections of two stems showing deviation of the pith related to possible stem breakage.

In some trees, KSR values showed large interannual fluctuations from the general trend (Fig. 5). The 3D reconstructions of the stem and knots for two of these trees showed large deviations of the pith of the main stem, likely a result of leader loss or stem damage. While one of these trees retained apical dominance in a single leader (T01), the other produced a fork (T09; Fig. 7). The model produced a good fit to all trees except tree T03, although visual examination of the 3D reconstruction of this stem revealed no obvious explanation for the lack of fit.

### Knot-level models

**Figure 8:**
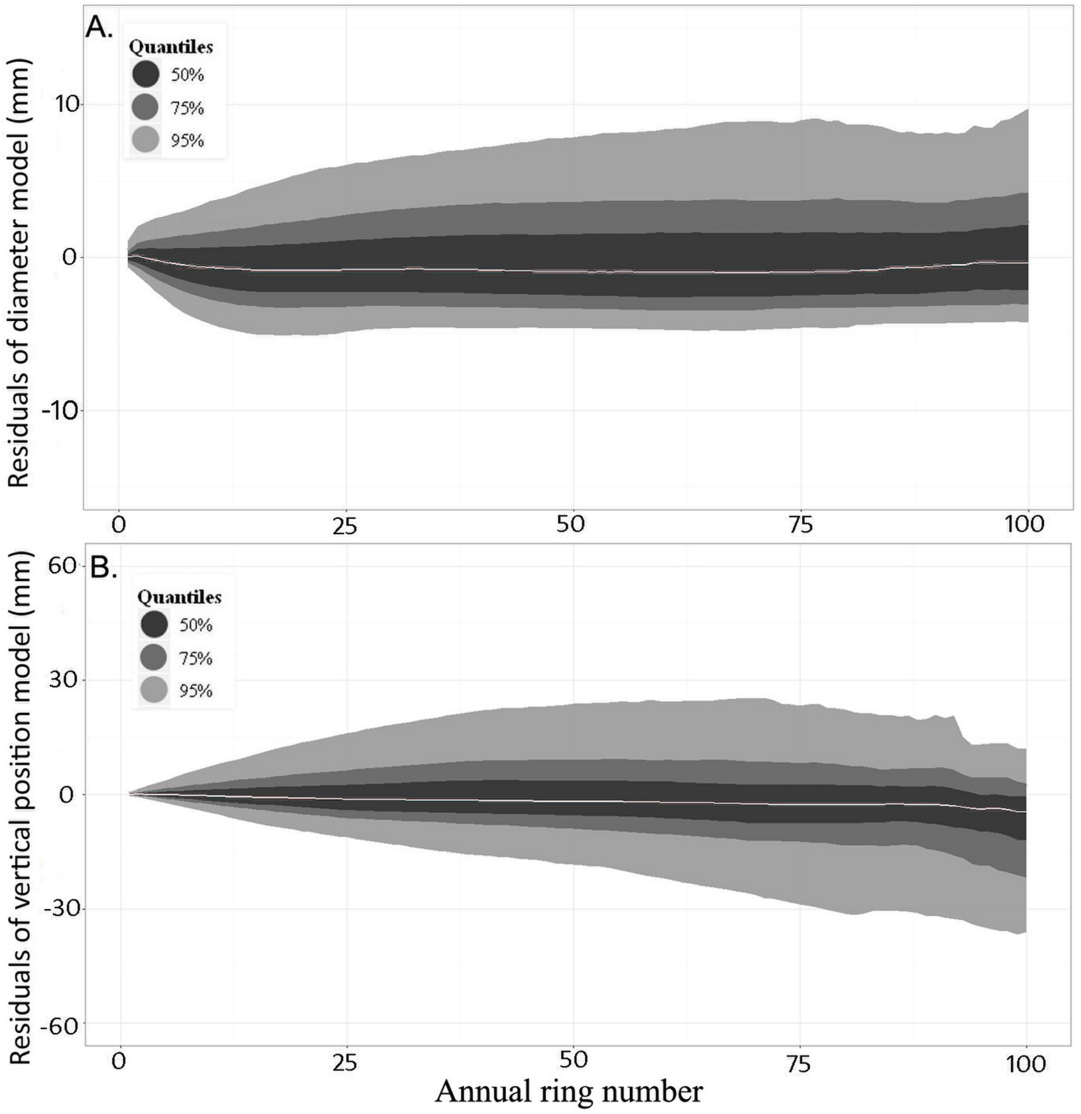
Distribution of the residuals (sorted by quantiles) against ring number when the model was applied to the evaluation dataset. (A) Knot diameter (Eq. (4) and Table 4) and (B) knot vertical position (Eq. (5) and Table 5). The grey line indicates the median of all observations for a given ring number. Contours provide the distribution around the median.

Table 4 shows the fixed effects parameter estimates and standard errors for each section of the final knot diameter model (Eq. (4)). To evaluate the model, knot diameter profiles were predicted and compared to observations in the evaluation dataset. Plots of the raw residuals (observed minus predicted values) showed that, on average, knot diameter was slightly underestimated in the middle section of the knot profiles, but overall the model was unbiased (Fig. 8A). The mean absolute error was 0.031 and the root mean square error (RMSE) 0.054. When the profile of each knot in the database was reconstructed by adding successive annual diameter predictions, the absolute value of 50% of the residuals was less than 2.6 mm along the pith-to-bark profiles, while the absolute value of 90% of the residuals was less than 9.7 mm.

**Table 4:** Fixed effects parameter estimates and standard errors for each section of the knot diameter model given by Eq. (4). Section 1, knot initiation (1–3 years); Section 2, growth phase (4–25 years); Section 3, stabilisation and death (>25 years). Section 1 predicts the diameter and sections 2 and 3 predict the diameter increment.

	Section 1			Section 2			Section 3		
Parameter	Estimate	S.E	*P*-value	Estimate	S.E	*P*-value	Estimate	S.E	*P*-value
*c*_1_				−0.0338	0.01127	0.0026	0.0139	0.00198	<0.0001
*c*_2_				0.5166	0.00219	<0.0001	0.9699	0.00150	<0.0001
*c*_3_	1.0144	0.00671	<0.0001	−0.0302	0.00047	<0.0001	−0.0020	0.00006	<0.0001
*c*_4_	0.3661	0.01665	<0.0001	0.1285	0.00508	<0.0001	0.0068	0.00058	<0.0001
*c*_5_							0.0002	0.00002	<0.0001
*c*_6_	0.2653	0.01055	<0.0001	0.1031	0.00094	<0.0001	0.0057	0.00053	<0.0001
*c*_7_				0.0549	0.00628	<0.0001			
*c*_8_				−0.0004	0.00011	0.0003	−0.0001	0.00002	<0.0001
*c*_9_	−0.0011	0.00029	<0.0001	−0.0004	0.00008	<0.0001	−0.0002	0.00001	<0.0001
*c*_10_							0.0006	0.00017	<0.0001

Table 5 shows the fixed effects parameter estimates and associated standard errors for each section of the final model of knot trajectory (Eq. (5)). Again, predictions of knot trajectory profiles were compared to observations in the evaluation dataset. On average, the model was unbiased along the knot profile up to ring 75, with a slight overestimation beyond this point (Fig. 8B). The mean absolute error for this model was 0.118 and the root mean square error (RMSE) 0.189. When the profile of each knot was reconstructed by adding successive annual trajectory predictions, the absolute value of 50% of the residuals was less than 11.9 mm along the entire pith-to-bark profiles, while the absolute value of 90% of the residuals was less than 36.7 mm.

**Table 5:** Fixed effects parameter estimates and standard errors for each section of the knot trajectory model given by Eq. (5). Section 1, typically increasing trajectory (years 0–50), Section 2, typically decreasing trajectory (years > 50).

	Section 1			Section 2		
Parameter	Estimate	S.E	*P*-value	Estimate	S.E	*P*-value
*d*_1_	−0.2753	0.03019	<0.0001	0.0188	0.00447	<0.0001
*d*_2_	−0.0027	0.00025	<0.0001	−0.0003	0.00014	0.0328
*d*_3_	0.1864	0.00236	<0.0001	0.9719	0.00391	<0.0001
*d*_4_	−0.0039	0.00012	<0.0001	0.0002	0.00004	<0.0001
*d*_5_	0.1294	0.00097	<0.0001	−0.0357	0.00255	<0.0001
*d*_6_	0.2498	0.00927	<0.0001	−0.0033	0.00149	0.0252
*d*_7_	0.0064	0.00211	0.0024			
*d*_8_	0.0036	0.00015	<0.0001			
*d*_9_	0.0009	0.00009	<0.0001	0.0001	0.00004	0.0074

### Simulations

**Figure 9:**
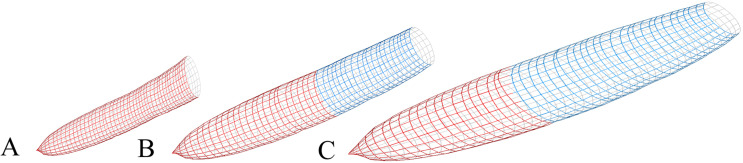
Simulations of a single knot from Eqs. (4) and (5) at 6.1 m of the main stem. Stem increments of tree T10 were used as the reference level for input parameters. (A) Radial growth decreased by 50%; (B) Reference level and (C) Radial growth increased by 50%. Real height growth from tree T10 was used for all simulations. The knot was assumed to have died when diameter increments reached zero. Red, live section; Blue, dead section

When we used the dimensions and growth of a real tree (T10) to simulate knot growth, the diameter increments in the early years of knot development were positively related to the radial growth of the main stem. However, knot longevity was reduced when the radial growth was artificially increased (and thus the HD ratio decreased). Knot growth ceased at ring 19 for the elevated growth scenario, but it was maintained along its entire profile (47 years) when stem growth was reduced (Fig. 9). In the real growth scenario, knot diameter increments began to decline around ring 25. Tree HD ratio also had a significant effect in the first section of the knot trajectory model, although the effect was only apparent in the lower stem (not shown).

**Figure 10:**
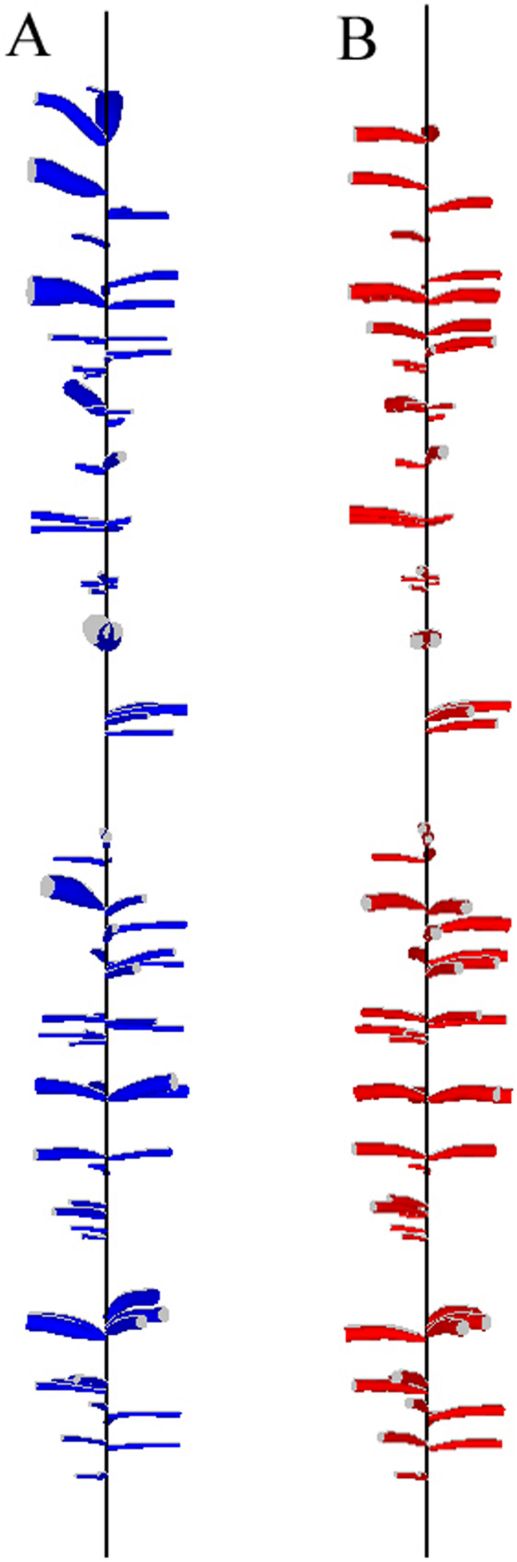
Reconstruction of a 1.5-m section from the base of the second log of tree T04 (i.e., at 2.5 m from the tree base). (A) Real knots extracted using the CT scanning data. (B) Simulated knots using the known insertion point, azimuthal orientation around the stem, and year of occlusion.

In the second simulation we reconstructed all knots in a 1.5-m section of tree T04. This showed that although the diameter of larger knots was slightly underestimated, the models generally produced accurate simulations of the diameter and shape of real knots. However, the models produced less variation in knot insertion angle than was observed in reality (Fig. 10), which would likely explain the larger residuals of the trajectory model.

## Discussion

### Resource allocation

This study provides further support to the idea that allocation of above-ground carbon assimilates in trees is directed towards locations where the potential return is the highest (Sprugel, 2002). To maintain a favourable position in the canopy, trees subjected to high levels of competition prioritize height growth over secondary radial growth (Lanner, 1985). Consequently, at a given age, the HD ratio is a useful predictor of assimilate partitioning among tree organs (West, 1993; King, 2005; McCarthy & Enquist, 2007). Despite large variation in annual knot growth, even among similar sized trees, the ratio of knot to stem area increment (KSR) was shown to decrease systematically with tree age. Similar ontogenetic effects have been highlighted by Wilson (1988) to describe changes in shoot: root ratio as a plant grows.

Under the assumption that stem or branch area increments are proportional to biomass accumulation, the observed correlation between KSR and HD indicates a shift in assimilate allocation towards branches when tree growth is constrained by competition. Likewise, Vincent (2006) found that lower light levels were associated with an increase in leaf life span, while King (1997) showed that the percentage of biomass allocated to branches was higher in understory seedlings than in those growing in large gaps. A similar concept of functional balance has also been used to explain the decrease in shoot:root ratio when soil nutrients are a limiting factor (Génard et al., 2007). Under the principles of teleonomy, these may be seen as adaptive responses of trees to environmental factors, which would optimize their growth and survival probability (Lacointe, 2000).

In this study, annual reconstructions of stem and branch development suggested that KSR values were also positively related to the number of new branches initiated in a growth unit. This is in agreement with the principles highlighted above, but it appears to contradict a common result of empirical branch distribution models, which is that vigorous trees tend to initiate more branches in a given year (Maguire, 1994; Mäkinen & Colin, 1999; Hein et al., 2007). However, these studies typically presented models for the number of nodal branches i.e., those forming a pseudo-whorl (Fisher & Honda, 1979). Furthermore, in models that consider both nodal and internodal branches, smaller branches (<5 mm) are usually ignored (Colin & Houllier, 1991; Auty et al., 2012). An advantage of using CT scanning technology is that all the knots were identifiable, including those that were occluded within the stems. Furthermore, the identification of annual growth units along the stem was made easier because it was possible to locate, with some certainty, the initiation point of branches at the stem’s pith (Duchateau et al., 2013b).

The relationship of knot growth to HD ratio could be clearly seen in the simulations of individual knot growth. An increase in HD ratio led to smaller but longer-lived knots. When coupled with our finding on branch initiation, this result is in agreement with the negative relationship between the number of branches and their size presented by West, Enquist & Brown (2009). Throughout the simulation, each knot was first located at the top of the stem but its position relative to the stem’s apex shifted as the tree grew in height. Therefore, in the slower growth scenario, the fact that the knot was still growing at the end of the simulation implies a slower rate of crown recession. A lower crown base in trees subject to high competition is consistent with previous results (Sprugel, 2002; Valentine et al., 2013) and offers further support for Milton’s Law of resource availability and allocation. Sprugel’s (2002) choice of name for this principle made reference to poet Milton’s (1667) phrase, “Better to reign in hell than serve in heaven.” He used this analogy to highlight the fact that although branches in light-favored conditions will tend to grow faster, a shaded branch on a shaded tree is more likely to survive and grow than a similarly-shaded branch on a dominant tree. Our model provides a time-series illustration of this principle. The vigorous growth of the knot in the first 10–15 years of the accelerated growth scenario suggests that the carbon budget of the branch was more positive than branches simulated in slow growth scenarios. Despite this, branch growth ceased earlier in the accelerated growth scenario. Clearly, such behaviour could not be predicted based on individual branch carbon budgets, which leads us to question the applicability of the branch autonomy principle when modelling branch growth.

### Modelling knot development

Previous studies have represented the dead portion of knots as a cylinder to reflect the cessation of growth (Björklund, 1997; Lemieux, Beaudoin & Zhang, 2001; Moberg, 2001). However, around 40% of knots in our sample data had declining diameter profiles in the outer stem, presumably as a result of branch deterioration after death. We accounted for this trend in the knot diameter model by allowing negative growth predictions (Fig. 9). The inclusion of the diameter and trajectory increments of the previous year as predictor variables allowed for smooth transitions between the knot sections, which provided realistic knot shapes. Furthermore, analysis of the model residuals showed that the models were relatively unbiased and generally accurate.

In the second simulation, annual predictions of knot diameter and trajectory produced realistic reconstructions of the real knot profiles using the known insertion point, orientation and year of occlusion of each knot. Models that can predict the vertical and azimuthal distribution of branches within a growth unit, as well as the initial insertion angle of each branch in the main stem, will provide even more realistic stem profiles. Even further improvements could be gained from the addition of a self-pruning sub-model (Mäkelä & Mäkinen, 2003).

The interpretation of our results on knot and stem allocation should therefore focus on general, long-term trends rather than on inter-annual variation. In fact, the long-term trends presented at the stem level should be more robust, since they aggregate information from a large number of individual knot profiles.

## Conclusion

This study has provided an improved representation of the internal structure of tree stems by linking knot development with stem growth. The use of CT scanning data allowed us to reconstruct knot and stem ontogeny with unprecedented detail over a substantial time period. We have found evidence for increased allocation to branches under conditions that limit the secondary growth of the stem, which indicates that branches are non-autonomous entities. We have also produced a model of individual knot morphology that could provide greater precision in the representation of knots in FSTMs, thus expanding their applicability to the wood processing sector.

## Additional Information and Declarations

### Data Deposition

The following information was supplied regarding the deposition of related data: DataDryad: https://datadryad.org/resource/doi:10.5061/dryad.kv028
